# Demographic and Clinical Characteristics of Early Travel-Associated COVID-19 Cases

**DOI:** 10.3389/fpubh.2020.573925

**Published:** 2020-12-23

**Authors:** Reham M. Marei, Mohamed M. Emara, Omar M. Elsaied, Gheyath K. Nasrallah, Tawanda Chivese, Hamad E. Al-Romaihi, Mohamed H. Althani, Asmaa A. Al Thani, Elmoubasher A. Farag, Hadi M. Yassine

**Affiliations:** ^1^Biomedical Research Center, Qatar University, Doha, Qatar; ^2^College of Medicine-QU Health, Qatar University, Doha, Qatar; ^3^College of Health Sciences-QU Health, Qatar University, Doha, Qatar; ^4^Public Health Department, Ministry of Public Health, Doha, Qatar

**Keywords:** coronavirus, traveler's risk to infection, clinical outcome, epidemiology, COVID-19

## Abstract

**Background:** SARS-CoV-2 continues to claim hundreds of thousands of people's lives. It mostly affects the elderly and those with chronic illness but can also be fatal in younger age groups. This article is the first comprehensive analysis of the epidemiological and clinical outcomes of the travel-associated SARS-CoV-2 cases until April 19, 2020.

**Methods:** Demographic and clinical data of travel-associated SARS-CoV-2 cases were collected for the period between January 16, 2020 and April 19, 2020. More than one hundred and eighty databases were searched, including the World Health Organization (WHO) database, countries' ministries websites, and official media sites. Demographic and clinical data were extracted and analyzed.

**Results:** A total of 1,186 cases from 144 countries meeting the inclusion criteria were reported and included in the analysis. The mean age of the cases was 44 years, with a male to female ratio of 1.6:1. Travel-associated cases originated from more than 40 countries, with China, Italy, and Iran reporting the highest numbers at 208, 225, and 155, respectively. Clinical symptoms varied between patients, with some reporting symptoms during the flights (117 cases; 9.87%). A total of 312 (26.31%) cases were hospitalized, of which 50 cases (4.22%) were fatal.

**Conclusion:** Major gaps exist in the epidemiology and clinical spectrum of the COVID-19 travel-associated cases due to a lack of reporting and sharing data of many counties. The identification and implementation of methodologies for measuring traveler's risk to coronavirus would help in minimizing the spread of the virus, especially in the next waves.

## Introduction

Out of seven coronaviruses known to infect humans, four are seasonal (229E, NL63, OC43, and HKU1) and cause common respiratory infections similar to influenza (1). Additionally, three novel coronaviruses of animal origin (zoonotic) have emerged in the last two decades and caused unprecedented outbreaks in human: the Severe Acute Respiratory Syndrome (SARS-CoV) that emerged in 2003 (2–4), the Middle East Respiratory Syndrome (MERS-CoV) that emerged in 2012 (5, 6), and the SARS-CoV-2 that emerged at the end of 2019 (7–10). All of the zoonotic viruses were associated with server respiratory illnesses.

SARS-CoV-2, which is the causative agent of Coronavirus disease 2019 (COVID-19), was first identified in Wuhan, China, in December 2019 (10). The virus then rapidly spread to other countries around the globe. The first ten countries which reported the virus after China were Thailand, Japan, South Korea, the USA, Singapore, Vietnam, Australia, France, and Malaysia (11, 12). The rapid spread of the virus to the different continents evoked WHO to declare the pandemic stage on March 11, 2020, making it the first zoonotic-origin coronavirus to reach this stage (13).

The clinical spectrum of COVID-19 ranges from asymptomatic/mild upper respiratory tract illness in about 80% of the patients to severe viral pneumonia with respiratory failure and even death. The major risk factors associated with disease severity include chronic illness, older age, and genetic dispositions. The incubation period of COVID ranges from 1 to 14 days (median 5 days), during which infected patients can transmit the virus even before experiencing any symptoms (7, 10, 14, 15).

The risk of acquiring an infectious agent during travel is relatively high. As per the 2015 records of the World Tourism Organization (WTO), 1.2 billion international tourist arrivals were documented (16). These figures are projected to increase further and reach 1.4 billion by 2020 and 1.8 billion by 2030, signifying the crucial public health impact that travel will have on global health security (17). Consequently, travelers represent a cohort of epidemiological importance because of the associated dynamicity, exposure to infectious diseases, and risk of circulating infections among populations. This concern has been further fueled by the rapid emergence and spread of various agents such as Ebola, MERS-CoV, Zika, and most recently, the SARS-CoV-2, although each of them is characterized by a different mode of transmission (18, 19).

Although MERS-CoV was identified in 2012, only 2,494 cases have been reported so far (20–25). Further, SARS-CoV-1 was contained in <2 years after affecting more than 8,000 cases, including 800 deaths (26, 27). As of October 15, 2020, SARS-CoV-2 had affected more than 40 millions worldwide, including at least one million reported deaths (fatality rate of about 2.5%), making it the most transmissible coronaviruses (zoonotic) identified so far (28). Although the significance of SARS-CoV-2 spread and transmission among populations is well recognized, there is a noticeable lack of the information about travel-associated cases. In this study, we report on SARS-CoV-2 travel-associated cases demography, clinical outcome, and risk factors associated with virus transmission.

## Methodology

The aim of this study was to analyze the epidemiological and clinical characteristics of the early SARS-CoV-2 cases or so-called travel-associated cases. Demographic and clinical data of travel-associated SARS-CoV-2 cases were collected for the period between January 16 and April 19, 2020. One hundred eighty-one cases databases were searched, including the World Health Organization (WHO) database, countries' ministries websites, and official media sites (Supplementary Table 1). A comprehensive data collection sheet of the individual study parameters was prepared using Microsoft Excel. The following information was extracted and summarized: age, gender, the origin of cases, occupation, destination, comorbidities, hospitalization, and clinical manifestation, and clinical outcome, symptoms appearance during travel, and the timeline for imported cases. Data were presented as medians, means, standard deviation (SD), percentage, and cumulative numbers. The data set was not complete for all reported cases, and hence, rates were calculated according to the available data. It is worth noting that we had collected the data to the best of our knowledge and capacity, relying on the resources listed in Supplemental Table 1.

## Results

### Demographic Characteristics

The demographic characteristics of the imported cases up to April 19, 2020 are described in Table 1. A total of 1,186 cases meeting the inclusion criteria were reported by that date. More than half of the data were missing for the age group (65.85%). People of all ages were affected, with a mean age of the cases being 44 (SD 18) years. The majority of cases were reported for those older than 20 years, while those <20 years of age represented only 3.20% of the documented cases. Higher cases were reported in males (27.82%) compared to females (17.37%), with a female to male ratio of (1:1.6).

**Table 1:** Demographic characteristics of travel-associated cases as of April 19 2020 (*n* = 1,186).

**Demographics**	**Criteria**	**Number (%)**
Age, years	Mean (SD)	44 (SD = 18)
Age, years	Median (IQR)	42
Age group, *n* (%)	0–20	38 (3.20)
	20–40	126 (10.62)
	40–60	143 (12.06)
	60–80	78 (6.58)
	>80	20 (1.69)
	Unspecified	781 (65.85)
Gender, *n* (%)	Female	206 (17.37)
	Male	330 (27.82)
	Not specified	650 (54.67)

### Cases Occupation

The cases had varying occupations as described in Table 2, with 1,143 cases occupation (96.37%) being unidentified. For the available data, students represented the highest percentage of (1.01%), followed by Doctors (0.84%) and Business staff (0.76%).

**Table 2:** Cases occupation (*N* = 1186).

**Occupation**	***N* (%)**
Student	12 (1.01)
Nurse	4 (0.34)
Business staff	9 (0.76)
Professor	2 (0.17)
Doctor	10 (0.84)
Music producer	1 (0.08)
Military staff	2 (0.17)
Office worker	3 (0.25)
Unspecified	1,143 (96.37)

#### Timeline of SARS-CoV-2 Spread Across the Globe

**Figure 1:**
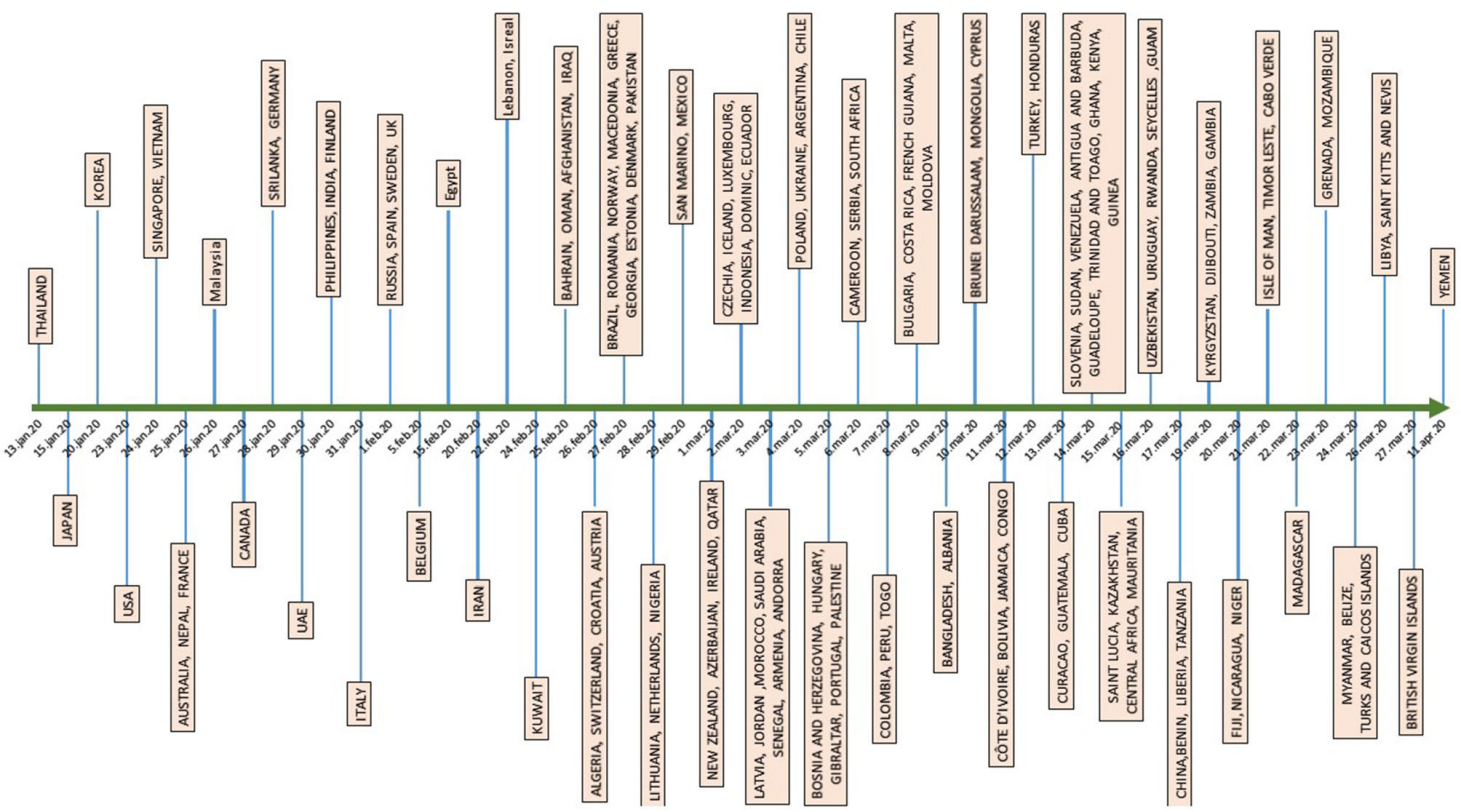
Timeline of SARS-CoV-2 spread to different countries between Jan 13 and April 11, 2020.

The timeline of SARS-CoV-2 spread to different countries between January 13 and April 11, 2020, is shown in Figure 1. The first ten countries to report the virus after China were: Thailand, Japan, South Korea, the USA, Singapore, Vietnam, Australia, France, and Malasia. Until February 27, 2020, China was the main hub for exporting the virus to 23 countries. After the mid-February, Italy and Iran became the epicenters for Europe and the Middle East, respectively. On March 17, China reported the first travel-associated case from abroad, reaching 264 cases by April 19, 2020. Imported cases per region and country are shown in Table 3.

**Table 3:** Travel-associated cases per region, country, and territory.

**Region, country, territory**	***N* (%)**
Western Pacific Region	421
Malaysia	11 (0.93)
New Zealand	3 (0.25)
China	264 (22.26)
Australia	33 (2.78)
Korea	14 (1.18)
Philippines	9 (0.76)
Singapore	32 (2.61)
Vietnam	26 (2.19)
Japan	24 (2.02)
Mongolia	2 (0.17)
Fiji	1 (0.08)
Brunei Darussalam	2 (0.17)
Territories	2
Guam	2 (0.17)
European Region	294
Spain	17 (1.43)
Italy	6 (0.50)
Germany	2 (0.17)
The United Kingdom	27 (2.28)
France	3 (0.25)
Turkey	2 (0.17)
Belgium	15 (1.26)
Netherlands	11 (0.93)
Switzerland	6 (0.50)
Portugal	6 (0.50)
Ireland	3 (0.25)
Austria	4 (0.34)
Sweden	13 (1.01)
Israel	10 (0.84)
Romania	19 (1.60)
Denmark	7 (0.59)
Norway	7 (0.59)
Serbia	1 (0.08)
Ukraine	1 (0.08)
Poland	4 (0.34)
Luxembourg	2 (0.17)
Finland	5 (0.42)
Hungary	1 (0.08)
Greece	5 (0.42)
Moldova	5 (0.42)
Croatia	5 (0.42)
Estonia	2 (0.17)
Azerbaijan	11 (0.93)
Slovenia	5 (0.42)
Lithuania	6 (0.50)
Uzbekistan	6 (0.50)
Bosnia& Herzegovina	6 (0.50)
Armenia	5 (0.42)
Kazakhstan	8 (0.67)
Andorra	1 (0.08)
Bulgaria	1 (0.08)
Kyrgyzstan	9 (0.76)
Albania	3 (0.25)
Macedonia	3 (0.25)
Iceland	13 (1.01)
Russia	5 (0.42)
Czech Republic	4 (0.34)
Cyprus	3 (0.25)
Latvia	4 (0.34)
Malta	8 (0.67)
Georgia	4 (0.34)
Territories	3
Isle of Man	1 (0.08)
Gibraltar	2 (0.17)
South-East Asia Region	79
Thailand	38 (3.20)
India	10 (0.84)
Indonesia	2 (0.17)
Bangladesh	3 (0.25)
Myanmar	2 (0.17)
Nepal	4 (0.34)
Timor-Leste	1 (0.08)
Taiwan	7 (0.59)
sir lanka	12 (1.01)
Eastern Mediterranean Region	151
UAE	10 (0.84)
Iran	4 (0.34)
Kuwait	42 (3.54)
Bahrain	10 (0.84)
Afghanistan	2 (0.17)
Oman	18 (1.52)
Iraq	18 (1.52)
Yemen	1 (0.08)
Lebanon	10 (0.84)
Qatar	7 (0.59)
Saudi Arabia	9 (0.76)
Tunisia	4 (0.34)
Jordan	1 (0.08)
Libya	1 (0.08)
Pakistan	8 (0.67)
Morocco	2 (0.17)
Sudan	1 (0.08)
Egypt	2 (0.17)
Djibouti	1 (0.08)
Territories	2
State of Palestine	2 (0.17)
Region of the Americas	153
USA	68 (5.73)
Canada	21 (1.77)
Brazil	4 (0.34)
Peru	6 (0.50)
Argentina	2 (0.17)
Chile	1 (0.08)
Colombia	3 (0.25)
Mexico	8 (0.67)
Bolivia	4 (0.34)
Costa Rica	1 (0.08)
Cuba	1 (0.08)
Dominican Republic	2 (0.17)
Ecuador	1 (0.08)
Uruguay	4 (0.34)
Guatemala	2 (0.17)
Venezuela	6 (0.50)
Jamaica	2 (0.17)
Trinidad and Tobago	3 (0.25)
Honduras	5 (0.42)
Antigua & Barbuda	3 (0.25)
Saint Kitts and Nevis	2 (0.17)
Grenada	1 (0.08)
Belize	1 (0.08)
Nicaragua	1 (0.08)
saint Lucia	1 (0.08)
Territories	8
French Guiana	3 (0.25)
Curacao	1 (0.08)
British Virgin Islands	2 (0.17)
Turks and Caicos Islands	1 (0.08)
Guadeloupe	1 (0.08)
African Region	72
Congo	5 (0.42)
Cameroon	1 (0.08)
Nigeria	4 (0.34)
Madagascar	8 (0.67)
South Africa	20 (1.69)
guinea	1 (0.08)
Ghana	4 (0.34)
Niger	1 (0.08)
Kenya	2 (0.17)
Algeria	2 (0.17)
Tanzania	1 (0.08)
Senegal	1 (0.08)
Togo	1 (0.08)
Liberia	1 (0.08)
Cabo Verde	1 (0.08)
Zambia	3 (0.25)
Rwanda	1 (0.08)
Benin	1 (0.08)
Central African Republic	1 (0.08)
Mozambique	2 (0.17)
Seychelles	2 (0.17)
Mauritania	8 (0.67)
Gambia	1 (0.08)
Côte d'Ivoire	1 (0.08)

### Origin of Travel-Associated Cases

A critical part of our analysis was to identify the origin of travel-associated cases. The majority of travel-associated cases originated from Italy 255 (21.50%), followed by China 208 (17.53%), Iran 155(13.07%), and Diamond Princess Cruise ship 141 (11.89%). Few other cases were reported from other countries, including the USA 72 (6.07%), UK, and France 46 (3.88%). Twenty-three subjects (1.94%) had missing data of the place of origin (Table 4).

**Table 4:** Origin of travel-associated cases (*N* = 1,186).

**Region, Country, Territory**	***N* (%)**
Western Pacific Region	231
China	208 (17.53)
Korea	4 (0.34)
Japan	8 (0.67)
Australia	4 (0.34)
Philippines	2 (0.17)
Singapore	2 (0.17)
Malaysia	3 (0.25)
European Region	507
Spain	44 (3.71)
Italy	255 (21.50)
Germany	23 (1.94)
France	46 (3.88)
Turkey	11 (0.93)
Belgium	22 (1.85)
Netherlands	2 (0.17)
Switzerland	18 (1.52)
Portugal	2 (0.17)
Ireland	3 (0.25)
Austria	19 (1.60)
Israel	2 (0.17)
UK	46 (3.88)
Russia	2 (0.17)
Czech Republic	1 (0.08)
Poland	1 (0.08)
Norway	2 (0.17)
Denmark	1 (0.08)
Greece	4 (0.34)
Hungary	2 (0.17)
Azerbaijan	1 (0.08)
South-East Asia Region	201
India	7 (0.59)
Indonesia	1 (0.08)
Thailand	1 (0.08)
Iran	155 (13.07)
Saudi Arabia	2 (0.17)
Pakistan	2 (0.17)
UAE	17 (1.43)
Qatar	1 (0.08)
Egypt	12 (1.01)
Morocco	1 (0.08)
Iraq	2 (0.17)
Oman	2 (0.17)
Region of the Americas	75
USA	72 (6.07)
Canada	1 (0.08)
Panama	1 (0.08)
Colombia	1 (0.08)
African Regions	8
South Africa	4 (0.34)
Burkina Faso	1 (0.08)
Mauritius	1 (0.08)
Senegal	4 (0.34)
Diamond Princess cruise ship	141 (11.89)
Unspecified	23 (1.94)

### Clinical Presentation and Symptoms

Most of the travel-associated cases presented with fever (45.87%), followed by cough (38.69%), flu-like symptoms (6.22%), sore throat (1.60%), and pneumonia (1.01%). Several cases (*n* = 117; 9.87%) reported symptoms during the flight from China. For the non-symptomatic cases on the flights (48; 4.05 %), symptoms were developed between 1 and 22 days after travel (mean = 7.4; Median = 7, SD = 4.63) (Table 5).

**Table 5:** Symptoms at presentation (*N* = 1,186).

**Symptoms**	**Number (%)**
Cough	460 (38.69)
Fever	544 (45.87)
Flu-like symptoms	74 (6.22)
pneumonia	12 (1.01)
Gastrointestinal tract	1 (0.08)
Sore throat	19 (1.60)
Unspecified	76 (6.40)
Symptomatic during flight	117 (9.87)
Non-symptomatic during flight	48 (4.05)
Nonspecific	1,021 (86.09)
Average days to develop symptoms after arrival	1–22 day
For days to develop symptoms	Mean = 7.4
For days to develop symptoms	Median = 7
For days to develop symptoms	SD = 4.63

### Comorbidities, Hospitalization, Severity, and Outcome of Travel-Associated Cases

One thousand ninety (91.91%) of the imported cases had unspecified data about former health conditions. About 32 (2.70 %) of the cases were apparently healthy. Comorbidities were reported in 96 (8.09 %) cases only. Comorbidities included hypertension (*n* = 19), cardiovascular diseases (*n* = 12), Diabetes mellitus (*n* = 12), cancer (*n* = 10), pulmonary diseases (*n* = 9) and kidney diseases (*n* = 2) (Table 6).

**Table 6:** Comorbidities in travel-associated cases (*N* = 1,186).

**Comorbidity diseases**	***N* (%)**
Hypertension	19 (1.60)
Cardiovascular disease	12 (1.01)
Diabetes mellitus	12 (1.01)
Pulmonary disease	9 (0.76)
Cancer	10 (0.84)
Kidney disease	2 (0.17)
Healthy	32 (2.70)
Unspecified	1,090 (91.91)

Hospitalization data were reported for 312 (26.31 %) cases. The majority were hospitalized because they “tested positive” for COVID19 infection (21.59%). Symptoms of COVID-19 was the second leading cause of hospitalization (2.95%), while some were hospitalized for developing pneumonia (1.69%). Other reasons for hospitalization are shown in Table 7.

**Table 7:** Reasons for hospitalization of travel-associated cases (*N* = 1,186).

**Reason**	***N* (%)**
Pneumonia	20 (1.69)
Tested positive (isolation)	256 (21.59)
Symptomatic	35 (2.95)
Already admitted due to a different condition	1 (0.08)
Unspecified	874 (73.69)

Out of 1,186 analyzed cases, 1,049 (88.45%) had a mild illness, and 135 (11.38%) were sever or critical cases. The fatality was reported in 50 (4.22%) cases (Table 8).

**Table 8:** Cases severity and outcome (*N* = 1,186).

**Case severity**	***N* (%)**
Mild	1,049 (88.45)
Sever	135 (11.38)
**CASE OUTCOME**	
Recovery	400 (33.73)
Death	50 (4.22)
Unspecified	736 (62.06)

## Discussion

Emerging infections continue to be a major threat to the human population. Several major outbreaks and pandemics have been reported in the past two decades due to emerging or re-emerging infections, including but not limited to the SARS outbreak in 2002 (Affecting >30 countries and ~9,000 people), pandemic H1N1 in 2009 (Worldwide; millions), MERS in 2012 (27 countries; ~2,500), Ebola in 2013 (10 countries; 30,000), and Zika in 2015 (87 countries; >100,000) (29–32).

The intrusion and spread of novel pathogens into the human population is attributed to several factors, majorly humans' behavior, social and environmental changes. In the past 20 years, six new coronaviruses emerged in the human population: SARS (2003), NL63 (2004), HKU1 (2005), MERS (2012), and SARS-CoV-2 (2019) (33). While SARS-CoV-1 was contained in <2 years, little is known about how the NL63 and HKU1 had emerged and spread in the human population. Both viruses (NL63 and HKU1) continue to circulate in humans, and they constitute up to 30% of the respiratory infections along with OC43 and 229E coronaviruses. However, we lack knowledge about the clinical manifestations in the primary patients who acquired these viruses. Unlike the other highly pathogenic coronaviruses, SARS-CoV-1, and MERS-CoV, only the SARS-CoV-2 reached the pandemic stage in a relatively short period (11). Within <11 months of its emergence, the virus affected more than 40 million, including more than one million confirmed deaths (12, 34).

Studies have shown that infected individuals with SARS-CoV-2 could spread the virus several days before clinical symptoms appear, and many of which do not even present any clinical manifestations (35). Accordingly, the risk of spread and transmission from these two groups is considered high. A recent modeling study suggests that asymptomatic persons might be the major drivers for the growth of the virus nationally and globally (36). This partially explains the late detection of the virus in China and, possibly, the reason for the widespread of the virus globally. Besides, a high viral load close to the onset of the symptoms suggests that the virus can be easily transmissible at an early stage of the infection (36).

The SARA-CoV-2 emerged in Wuhan, China, in December 2019. By February 1, 2020, the virus was reported in Thailand, Japan, South Korea, USA, Vietnam, Singapore, Australia, France, Nepal, Malaysia, Canada, Cambodia, Srilanka, Germany, United Arab Emirates, Philippines, India, Finland, and Italy (chronological order) (11, 12). Italy and Iran reported the first cases on January 31 and February 20, becoming the epicenter for Europe and the Middle East, respectively. By February 26, 2020, the virus massively spread to many countries, resulting in declare of the pandemic on March 11, 2020.

Although the reproduction number (R_0_) for SARS-CoV-1 and SARS-CoV-2 is relatively similar (24), the containment measures were only efficient against the earlier virus. Many factors could have contributed to the rapid spread of SARS-CoV-2, including host, viral and environmental factors. Although travel, specifically air travel, is a significant player in infectious diseases spread across countries, limited studies have been conducted in this domain. Here, we summarize the main epidemiological and clinical features of travel-associated COVID-19 up to April 19, 2020.

Travel-related introduction and tourism-related spread contributed substantially to the transmission of the virus across and within countries during the early phase of the COVID-19 pandemic. After an extensive search of national and international databases (Supplementary Table 1), we reported data from 145 countries and territories. Unfortunately, not all clinical and epidemiological data were available for all cases, which highlights the importance and the need for a better recording system, supervised by WHO and other international organizations, to preserve and share data.

In the absence of other studies on travel-associated CoV illness, our analysis is limited but remains essential for understanding the early phase of the pandemic. The mean age of the COVID-confirmed cases in our study was 44 (Range: 1–88), compared to 39.9 and 56 for MERS and SARS, respectively. On the other hand, the male to female ratio was calculated at 1.6:1 compared to 3.3:1 and 1:1.3 for MERS and SARS, respectively (37, 38). Accordingly, our data suggest that at the early stages of the outbreak, COVID-19 affected more males than females, similar to MERS, but younger age groups like SARS. According to several studies, age is considered a major risk factor for severe diseases (9, 14). Hence, the relatively young population (Mean of 44) of travelers could have been asymptomatic carriers who spread the virus across the globe. It is worth noting that most of the SARS and MERS cases were reported in healthcare or closed settings (21, 26, 38). The occupation information was missing for most of the travel-associated cases in our study. Healthcare workers (nurses and doctors) and students reported 1.18 and 1% of the occupations according to the available data. All other occupations represented proportions <1%.

The major obstacle of our analysis was the difficulty of finding reliable data from several countries. This limitation was noticeable in both developed and developing countries, indicating a lack of proper response to the crisis since the early stages. For example, the United States and Italy were among the earliest countries to report cases. Still, data about the travel-associated illness was very scarce. This partially explains the tremendous increase in cases at later stages in both countries. On the other hand, most of the organized and informative data were collected from Thailand, Malaysia, and South Korea, which had better control of the outbreak. This again emphasizes the importance of sharing data and making it available for public health officials and experts in the field to assess the problem and undertake the proper measures.

Initially, most of the travel-associated cases originated from China; however, Italy and Iran became the two main epicenters for Europe and The Middle East, respectively. Interestingly, between February 24 and March 13, China reported 264 imported cases from 17 countries. Further, the origin of travel-associated cases was identified in more than 40 countries during the period of the study, indicating the easiness of virus spread regardless of all implemented containment measures.

Identifying the incubation period and onset of symptoms in patients is very critical while preparing guidelines and plans to battle the pandemic. Unfortunately, this essential information was missing for most of the cases in our study. In general, patients presented a variety of symptoms, similar to what has been reported in other studies (8, 10, 39). Interestingly, 117 of the travel-associated cases presented symptoms during the flight from China. These were traveling to Singapore, Malaysia, Thailand, South Korea, Philippines, Germany, Vietnam, and Iran. On the other hand, data was available for 48 cases who developed symptoms up to 22 after travel. Accordingly, the implemented quarantine period of 14 days might be a little short and perhaps requires further consideration.

On a related aspect, it is essential to study viral survival on airplanes furniture, filters, and other parts, and its suspension as an aerosol during the flight. During the SARS-CoV-1 outbreak, studies had investigated the spread of the virus among passengers who traveled on 40 flights carrying symptomatic patients (40). Transmission seems to have occurred on board of only five out of the 40 flights, with only one reporting major spread. In one flight, a 72-year-old man, infected with SARS-CoV-1, transmitted the virus to 22 other passengers, including two aircraft crew (40, 41). Infection in the passengers was related to the physical proximity to the index patient. On the contrary, in another flight that carried four symptomatic patients, only one patient got infected. Few studies had reported on probable aircraft transmission of SARS-CoV-2 (42), and further studies on this aspect are needed. Most of the airlines had suspended their international flights, which affected the aviation economy globally (43, 44). This situation might last for an extended period until a herd immunity is achieved via national infection or vaccination. Until then, the airline companies are preparing a new set of guidelines that enable them to operate while maintaining a safe environment for passengers, including social distancing and other measures in the airports and onboard.

In about 8 months (November 2002–July 2003), the SARS-CoV-1 reached 31 counties, most of which (23; 75%) reported <10 cases (32). In more than 8 years, MERS-CoV was reported in 27 countries (23), with major outbreaks happened in Saudi Arabia and South Korea in hospital settings. Interestingly, MERS-CoV reported minimal spreading during crowd seasons in Saudi Arabia during Haj and Umra (38). SARS-CoV-2, on the other hand, reached all over the globe and spread rapidly in communities in a very short period. Accordingly, the three zoonotic CoV seems to follow a different trend of human-to-human transmission and spread.

Travel has led to the introduction and spread of SARS-CoV-2 in several ways, mainly through the mobility of individuals during trips (airports, airplanes, ships, and others), or afterward through family and social gatherings. Standard non-pharmaceutical measures are the most critical approaches for controlling the spread of COVID-19 in all settings, including during travel. Such measures include physical distancing, hand hygiene, respiratory etiquette, as well as other infection prevention and control measures. Information about the risk and symptoms of COVID-19, and advice to avoid travel while experiencing any of the COVI-19 related symptoms is essential (36, 45).

The SARS-CoV-2 emerged during the wintertime of temperate regions, which could have eased the spread of the virus. Interestingly, the virus spread did not slow down during the summertime, and it continues to surge in multiple countries. More flights are now in motion, easing the re-introduction of the virus to countries that might have controlled the spread on infection. This becomes more worrisome if the virus mutates to become more pathogenic and transmissible. Accordingly, several measures have to be implemented to reduce virus transmission with travelers, including airport screening, quarantine of contacts, and isolation of confirmed cases after travel. More importantly, WHO and other international organizations should urge countries to report in detail all the epidemiological and clinical characteristics of identified travel-associated cases, along with the status of transmission on all the flights. Proper decontamination of aircraft and social distancing in the flight might reduce the transmission, and hence, the control of the virus spread internationally.

## Data Availability Statement

The original contributions presented in the study are included in the article/Supplementary Material, further inquiries can be directed to the corresponding author/s.

## Author Contributions

ME, TC, EF, and HY designed the concept. RM and OE collected and categorized data. RM analyzed data and generated tables and figures. AA provided funding. HY wrote the first draft. All authors revised the draft and agreed on the final version of the paper.

## Conflict of Interest

The authors declare that the research was conducted in the absence of any commercial or financial relationships that could be construed as a potential conflict of interest.
