# Supplementary material for: Demographic and Clinical Characteristics of Early Travel-Associated COVID-19 Cases
Source: Front Public Health. 2020 Dec 23;8:573925. doi: 10.3389/fpubh.2020.573925 (PMC7786434; doi:10.3389/fpubh.2020.573925)
Supplement: Supplementary file 1 [file Data_Sheet_1.PDF]

Country	Link References
Afghanistan	https://www.dhis2.org/covid-19
	http://covid.moph-dw.org/#/
	https://reliefweb.int/updates?search=Afghanistan%20covid19&page=4
Albania	https://coronavirus.al/
	https://coronavirus.al/category/lajme/
	https://coronavirus.al/statistika/
Algeria	http://covid19.sante.gov.dz/
Andorra	www.govern.ad
	https://www.govern.ad/comunicats?start=112
	https://www.govern.ad/coronavirus
Antigua and Barbuda	www.covid19.gov.ag
	https://ab.gov.ag/detail_page.php?page=42.
Argentina	www.argentina.gob.ar
	https://www.argentina.gob.ar/coronavirus/informes-diarios/reportes
	https://www.argentina.gob.ar/coronavirus/informes-diarios/sala-de-situacion
Armenia	www.ncdc.am
	https://ncdc.am/coronavirus/confirmed-cases-by-days/
	https://ampop.am/covid19-coronavirus-dynamic-statistics-in-armenia/
	https://twitter.com/ampoparmenia
	https://ampop.am/covid-19-deaths-in-armenia/
Australia	www.health.gov.au
	https://www.health.gov.au/resources/publications/coronavirus-covid-19-at-a-glance-5-april-2020
	https://www.health.gov.au/news/health-alerts/novel-coronavirus-2019-ncov-health-alert/coronavirus-covid-19-current-situation-and-case-numbers
	https://www.health.gov.au/news/health-alerts/novel-coronavirus-2019-ncov-health-alert/coronavirus-covid-19-news-and-media?page=9
Austria	www.bmi.gv.at
	https://www.ots.at/pressemappe/54/bundesministerium-fuer-inneres/seite/25
	https://info.gesundheitsministerium.at/?l=en
Azerbaijan	www.koronavirusinfo.az
	https://koronavirusinfo.az/az/page/xeberler?category=33&page=30
Bahrain	www.moh.gov.bh
	https://www.moh.gov.bh/COVID19/News
	https://www.moh.gov.bh/COVID19/ContactTracing1
Bangladesh	www.iedcr.gov.bd
	https://old.iedcr.gov.bd/website/index.php/component/content/article/11-others/227-pressrelease
	https://iedcr.gov.bd/covid-19/covid-19-situation-updates
Belgium	https://covid-19.sciensano.be/fr
	https://covid-19.sciensano.be/fr/covid-19-situation-epidemiologique
Belize	http://health.gov.bz/www/
	https://m-o-h.maps.arcgis.com/apps/opsdashboard/index.html#/be04bd9ca6984ecdb1980568cef26920
	https://www.facebook.com/GOBPressOffice/
Benin	www.gouv.bj

	https://www.gouv.bj/coronavirus/flashinfos/
	https://www.gouv.bj/coronavirus/articles/
Bolivia	https://www.minsalud.gob.bo/
	https://www.minsalud.gob.bo/centro-de-prensa/notas-de-prensa?start=760
Bosnia and Herzegovina	http://www.mcp.gov.ba/?lang=en
Brazil	https://coronavirus.saude.gov.br/
	https://susanalitico.saude.gov.br/extensions/covid-19_html/covid-19_html.html
	https://covid.saude.gov.br/
	http://www.brazil.gov.br/government/official-notes/press-release-1
British Virgin Islands	https://bvi.gov.vg/covid-19
	https://bvi.gov.vg/press-release?title=&field_tags_tid%5B0%5D=1732&field_date_of_news_item_value%5Bmin%5D%5Bdate%5D=&field_date_of_news_item_value%5Bmax%5D%5Bdate%5D=&page=24
Brunei Darussalam	http://www.moh.gov.bn/SitePages/COVID-19.aspx
	http://www.moh.gov.bn/SitePages/pressreleaseCOVID-19.aspx
Bulgaria	www.coronavirus.bg
	https://coronavirus.bg/bg/statistika
	https://coronavirus.bg/bg/news?p=47
Cabo Verde	www.covid19.cv
	https://covid19.cv/category/boletim-epidemiologico/page/12/
Cameroon	https://www.minsante.cm/site/?q=en/epid-mie-de-coronavirus-covid---19-
Canada	https://www.canada.ca/en/public-health/services/diseases/coronavirus-disease-covid-19.html
	https://www.canada.ca/en/public-health/services/diseases/coronavirus-disease-covid-19/epidemiological-economic-research-data.html
	https://www150.statcan.gc.ca/t1/tbl1/en/tv.action?pid=1310078101
	https://health-infobase.canada.ca/covid-19/epidemiological-summary-covid-19-cases.html
Central African Republic	https://www.afro.who.int/health-topics/coronavirus-covid-19
	https://www.afro.who.int/sites/default/files/COVID-19%20situation%20reports/COVID-19-Bulletin_18%20Feb.pdf
	http://whotogo-whoafroccmaster.newsweaver.com/JournalEnglishNewsletter/1fyxy4tt73e
Channel Islands	https://www.gov.je/health/coronavirus/Pages/index.aspx
	https://www.gov.je/Health/Coronavirus/Pages/CoronavirusCases.aspx
Chile	https://www.minsal.cl/23-informe-epidemiologico-covid-19/
	https://www.minsal.cl/nuevo-coronavirus-2019-ncov/informe-epidemiologico-covid-19/
China	https://www.coronavirus.gov.hk/eng/index.html
	https://chp-dashboard.geodata.gov.hk/covid-19/en.html
	https://www.news.gov.hk/eng/categories/covid19/index.html
Colombia	www.coronaviruscolombia.gov.co
	https://coronaviruscolombia.gov.co/Covid19/index.html
Congo	https://www.afro.who.int/health-topics/coronavirus-covid-19
	http://whotogo-whoafroccmaster.newsweaver.com/JournalEnglishNewsletter/1fyxy4tt73e
	https://www.afro.who.int/sites/default/files/COVID-19%20situation%20reports/COVID-19-Bulletin_18%20Feb.pdf
Costa Rica	https://www.ministeriodesalud.go.cr/index.php
	https://www.ministeriodesalud.go.cr/index.php/centro-de-prensa/noticias/741-noticias-2020

	https://www.ministeriodesalud.go.cr/index.php/centro-de-prensa/noticias/741-noticias-2020/1725-situacion-nacional-covid-19
	https://www.ministeriodesalud.go.cr/index.php/centro-de-prensa/noticias/741-noticias-2020/1532-lineamientos-nacionales-para-la-vigilancia-de-la-infeccion-por-coronavirus-2019-ncov
Croatia	www.koronavirus.hr
	https://www.koronavirus.hr/najnovije/34?&=&page=26
Cuba	https://salud.msp.gob.cu/
	https://twitter.com/intent/follow?original_referer=https%3A%2F%2Fsalud.msp.gob.cu%2F%3Flang%3Den&partner=tfwp&ref_src=twsrc%5Etfw&screen_name=japortalmiranda&tw_p=followbutton
	https://twitter.com/PresidenciaCuba?ref_src=twsrc%5Etfw%7Ctwcamp%5Eembeddedtimeline%7Ctwterm%5Eprofile%3APresidenciaCuba&ref_url=https%3A%2F%2Fsalud.msp.gob.cu%2F
	https://twitter.com/DiazCanelB?ref_src=twsrc%5Etfw%7Ctwcamp%5Eembeddedtimeline%7Ctwterm%5Eprofile%3ADiazCanelB&ref_url=https%3A%2F%2Fsalud.msp.gob.cu%2F
Curacao	www.curacaochronicle.com
Cyprus	https://www.moh.gov.cy/moh/moh.nsf/All/0D5A0919CACA4BF8C225851B003E098C
	https://www.coronavirus.mlsi.gov.cy/blog
	https://www.pio.gov.cy/coronavirus/en/press.html
Czech	https://onemocneni-aktualne.mzcr.cz/covid-19
	https://onemocneni-aktualne.mzcr.cz/covid-19/prehledy-khs
Denmark	www.sst.dk
	https://www.sst.dk/da/corona/Status-for-epidemien/Sundhedsstyrelsens-statusrapporter
Djibouti	https://sante.gouv.dj/
	https://covid19.gouv.dj/statistic
	https://covid19.gouv.dj/
	https://sante.gouv.dj/storage/publications/August2020/7Hkat6Oi4LXDIyKm95qf.pdf
	https://sante.gouv.dj/storage/publications/April2020/cMcQEVZc9fS4wLjvUSqA.pdf
Dominican Republic	www.msp.gob.do
	https://www.msp.gob.do/web/?s=coronavirus&paged=47
	https://www.msp.gob.do/web/?page_id=6948#1586785071781-f0e8c057-f5f4
Ecuador	https://www.salud.gob.ec/coronavirus-covid-19/
	https://www.salud.gob.ec/boletines-epidemiologicos-coronavirus-por-semanas/
Egypt	https://www.care.gov.eg/default.html
Estonia	www.terviseamet.ee
	https://www.terviseamet.ee/et/uudised?page=26
Fiji	http://www.health.gov.fj/media-center/covid-19/
	http://www.health.gov.fj/media-center/novel-coronavirus-covid-19/media-releases/
Finland	https://stm.fi/en/frontpage
	www.experience.arcgis.com
	www.fbcnews.com.fj
	https://stm.fi/en/press-releases?p_p_id=com_liferay_asset_publisher_web_portlet_AssetPublisherPortlet_INSTANCE_WEbF8vffQStC&p_p_lifecycle=0&p_p_state=normal&p_p_mode=view&_com_liferay_asset_publisher_web_portlet_AssetPublisherPortlet_INSTANCE_WEbF8vffQStC_delta=20&p_r_p_resetCur=false&_com_liferay_asset_publisher_web_portlet_AssetPublisherPortlet_INSTANCE_WEbF8vffQStC_cur=10
	https://stm.fi/documents/1271139/21475529/COVID+19+-epidemian+hallinnan+hybridistrategian+toteuttaminen+%E2%80%93+Mahdolliseen+toiseen+aaltoon+varautuminen.pdf/3c667955-2927-5067-8ca8-e51d79796d8e/COVID+19+-

	epidemian+hallinnan+hybridistrategian+toteuttaminen+%E2%80%93+Mahdolliseen+toiseen+aaltoon+varautuminen.pdf?t=1598343908408
France	https://solidarites-sante.gouv.fr/
	www.santepubliquefrance.fr
	https://www.data.gouv.fr/en/dashboard/
	https://solidarites-sante.gouv.fr/soins-et-maladies/maladies/maladies-infectieuses/coronavirus/etat-des-lieux-et-actualites/article/points-de-situation-janvier-fevrier-mars-2020
Estonia	www.terviseamet.ee
	https://www.terviseamet.ee/et/uudised?page=23
French Guiana	www.guyane.gouv.fr
	http://www.guyane.gouv.fr/Politiques-publiques/COVID-19/Covid-19-Points-de-situation/Point-de-situation-n-1-5-cas-averes
Gambia	https://www.afro.who.int/countries/827/news
	https://www.who.int/countries/gmb/
Georgia	www.stopcov.ge
	https://dph.georgia.gov/covid-19-daily-status-report
Germany	https://interaktiv.tagesspiegel.de/lab/sars-cov-2-das-virus-in-echtzeit/
	https://www.rki.de/DE/Content/InfAZ/N/Neuartiges_Coronavirus/Situationsberichte/Gesamt.html
Ghana	www.ghanahealthservice.org
	https://www.ghanahealthservice.org/covid19/
Gibraltar	https://www.gibraltar.gov.gi/health
	https://www.gibraltar.gov.gi/press/covid-19-press-releases?np=19
Greece	www.moh.gov.gr
	https://www.moh.gov.gr/articles/ministry/grafeio-typoy/press-releases?page=18
Grenada	https://www.gov.gd/
	https://covid19.gov.gd/grenadas-first-covid-19-patients-condition-worsens/
Guadeloupe	www.guadeloupe.gouv.fr
	http://www.guadeloupe.gouv.fr/Politiques-publiques/Risques-naturels-technologiques-et-sanitaires/Securite-sanitaire/Informations-coronavirus/Les-communiques-de-presse/Les-communiques-de-presse-de-la-prefecture
Guatemala	www.mspas.gob.gt
	https://tablerocovid.mspas.gob.gt/
Guinea	www.anss-guinee.org
	https://anss-guinee.org/welcome/news/5
Honduras	www.covid19honduras.org
	https://www.covid19honduras.org/?q=Comunicados&page=29
Hungary	www.koronavirus.gov.hu
	https://www.facebook.com/koronavirus.gov.hu
	https://koronavirus.gov.hu/hirek?page=159
Iceland	www.covid.is
	https://www.covid.is/tolulegar-upplysingar
	https://www.covid.is/tilkynningar
India	https://www.mohfw.gov.in/
	http://dhs.kerala.gov.in/advisories/
Indonesia	www.covid19.go.id

	https://covid19.go.id/peta-sebaran
	https://covid19.go.id/p/berita?page=96
Iran	https://en.irna.ir/
	https://en.irna.ir/page/search.xhtml?q=coronavirus&a=0&alltp=true&allpl=true&pi=117&allsr=true&pageSize=20&allty=true
Iraq	emro.who.int/irq/iraq-news/ministry-of-health-of-iraqwho-launch-global-report-on-disability.html
Ireland	www.gov.ie
	https://www.gov.ie/en/publication/ce3fe8-previous-updates-on-covid-19-coronavirus/
	https://www.gov.ie/en/publication/72d92-updates-on-covid-19-coronavirus-from-april-june-2020/
Isle of Man	https://covid19.gov.im/
	https://covid19.gov.im/news-releases-statements/?Page=13&
Israel	https://govextra.gov.il/ministry-of-health/corona/corona-virus-en/
	https://www.gov.il/en/departments/news/?OfficeId=104cb0f4-d65a-4692-b590-94af928c19c0&skip=260&limit=270
Italy	http://www.salute.gov.it/portale/p5_11.jsp
	http://cerca.ministerosalute.it/search?ulang=it&proxystylesheet=comunicatiPORT_front-end&access=p&sort=date%3AD%3AL%3Ad1&wc=200&ud=1&entqr=3&output=xml_no_dtd&filter=p&q=coronavirus&site=comunicatiPORT_collection&wc_mc=1&oe=UTF-8&tlen=2048&getfields=*&client=comunicatiPORT_front-end&ie=UTF-8&entqrm=0&start=120
	http://cerca.ministerosalute.it/search?ulang=it&proxystylesheet=notizieNASPORT_front-end&access=p&sort=date%3AD%3AL%3Ad1&wc=200&ud=1&entqr=3&output=xml_no_dtd&filter=p&q=coronavirus&site=newsNASPORT_collection&wc_mc=1&oe=UTF-8&tlen=2048&getfields=*&client=notizieNASPORT_front-end&ie=UTF-8&entqrm=0&start=30
Jamaica	https://jamcovid19.moh.gov.jm/
	https://admin.jamcovid19.moh.gov.jm/public/storage/resources/PressRelease.pdf
Japan	www.mhlw.go.jp
	https://www.mhlw.go.jp/english/new-info/index.html#Jan
Jordan	www.corona.moh.gov.jo
	https://corona.moh.gov.jo/ar/MediaCenter?page=18
Kazakhstan	www.hls.kz
	https://hls.kz/%D0%BA%D0%BE%D1%80%D0%BE%D0%BD%D0%B0%D0%B2%D0%B8%D1%80%D1%83%D1%81%D0%BD%D0%B0%D1%8F-%D0%B8%D0%BD%D1%84%D0%B5%D0%BA%D1%86%D0%B8%D1%8F-covid-19
	https://www.coronavirus2020.kz/
Kenya	https://www.health.go.ke/
	https://www.health.go.ke/press-releases/
Kuwait	https://corona.e.gov.kw/
	https://corona.e.gov.kw/Ar/Home/BreakingNews
Kyrgyzstan	www.med.kg
	http://www.med.kg/en/news.html?start=270
Latvia	https://arkartassituacija.gov.lv/
	https://www.spkc.gov.lv/lv/jaunumi?category%5B179%5D=179&category%5B60%5D=60&created%5Bmin%5D=--.--.----&created%5Bmax%5D=--.--.----&page=11
Lebanon	https://corona.ministryinfo.gov.lb/
	https://corona.ministryinfo.gov.lb/news/list/2?page=429
Liberia	www.frontpageafricaonline.com
	https://frontpageafricaonline.com/category/health/page/21/

Libya	www.covid19.ly
	https://www.facebook.com/NCDC.LY/?hc_ref=ARTJ4YaskzlRqMlxM-TqWYKQRJs3W0e6KCRJTDdplaiCPv4vHOCqVs8rV5hj-juXYTw&ref=nf_target&fref=tag&__tn__=kC-R
Lithuania	www.koronastop.lrv.lt
	https://koronastop.lrv.lt/lt/naujienos?page=79
Luxembourg	www.msan.gouvernement.lu
	https://coronavirus.gouvernement.lu/fr/communications-officielles.html?b=548
Madagascar	https://mg.usembassy.gov/u-s-citizen-services/security-and-travel-information/covid-19-information/
	https://www.cdc.gov/coronavirus/2019-ncov/covid-data/covidview/covid-view-past-summaries.html?Sort=Article%20Date%3A%3Adesc&Page=3
	https://madagascar.co.uk/coronavirus
Magnolia	www.ikon.mn
Malaysia	https://www.moh.gov.my/
	https://www.moh.gov.my/index.php/pages/view/349?mid=29
Malta	https://deputyprimeminister.gov.mt/en/Pages/health.aspx
Mauritania	www.alakhbar.info
Mexico	www.coronavirus.gob.mx
Moldova	https://msmps.gov.md/en
	https://ansp.md/index.php/category/actualizarea-situatiei-privind-coronavirus/page/30/
Morocco	http://www.covidmaroc.ma/Pages/AccueilAR.aspx
Mozambique	www.covid19.ins.gov.mz
Myanmar	https://www.mohs.gov.mm/
	https://mohs.gov.mm/page/9575
Nepal	www.covid19.mohp.gov.np
Netherlands	www.rivm.nl
	https://www.rivm.nl/coronavirus-covid-19/grafieken
	https://www.rivm.nl/coronavirus-covid-19/archief-corona-updates#februari-2020-486401-more
New Zealand	www.health.govt.nz
	https://www.health.govt.nz/news-media/media-releases?page=29
Nicaragua	https://www.who.int/countries/nic/
Niger	www.coronavirus.ne
Nigeria	https://covid19.ncdc.gov.ng/
Norway	https://www.regjeringen.no/en/dep/hod/id421/
	https://www.regjeringen.no/en/topics/koronavirus-covid-19/timeline-for-news-from-norwegian-ministries-about-the-coronavirus-disease-covid-19/id2692402/
Oman	moh.gov.om/en/home
	https://www.moh.gov.om/en/-59
Pakistan	www.covid.gov.pk
Peru	https://www.gob.pe/minsa/
	https://www.gob.pe/busquedas?institucion[]=minsa&reason=sheet&sheet=1&term=coronavirus
Philippines	https://www.doh.gov.ph/
	https://www.doh.gov.ph/2019-ncov?page=51
Poland	www.gov.pl
	https://www.gov.pl/web/koronawirus/dzialania-rzadu?page=4&size=10
Portugal	www.covid19.min-saude.pt

Qatar	https://covid19.moph.gov.qa/EN/Pages/default.aspx
Republic of North Macedonia	www.zdravstvo.gov.mk
	http://zdravstvo.gov.mk/sq/category/soopshtenija-2/page/15/
Reunion	www.reunion.gouv.fr
	http://www.reunion.gouv.fr/points-de-situation-r464.html?debut_articles_rub=200#pagination_articles_rub
Romania	www.stirioficiale.ro
	https://stirioficiale.ro/informatii?page=70
Russia	https://www.rosminzdrav.ru/en
	https://minzdrav.gov.ru/news?start=2020-01-01&end=2020-10-04
Rwanda	https://moh.gov.rw/index.php?id=188
	https://www.rbc.gov.rw/index.php?id=727
Saint Kitts and Nevis	https://www.covid19.gov.kn/
Saint Lucia	www.covid19response.lc
Saudi Arabia	https://www.moh.gov.sa/en/Ministry/MediaCenter/Publications/Pages/covid19.aspx
Senegal	www.sante.gouv.sn
	http://www.sante.gouv.sn/actualites?page=23
Serbia	www.covid19.rs
Seychelles	www.health.gov.sc
	http://www.health.gov.sc/index.php/covid-19/press-update/page/4/
Singapore	www.moh.gov.sg
	https://www.moh.gov.sg/covid-19/past-updates
Slovenia	www.covid-19.sledilnik.org
South Africa	www.sacoronavirus.co.za
South Korea	www.cdc.go.kr
	https://www.cdc.go.kr/board/board.es?mid=a20501000000&bid=0015
South Sudan	www.radiotamazuj.org
	https://radiotamazuj.org/en/news?startdate=&enddate=&search=coronavirus&page=18
Spain	www.mscbs.gob.es
	https://www.mscbs.gob.es/profesionales/cargarNotas.do?time=1577833200000
Sri Lanka	www.epid.gov.lk
	http://www.epid.gov.lk/web/index.php?lang=en
State of Palestine	www.corona.ps
Sudan	https://www.who.int/workforcealliance/countries/sdn/en/
Sweden	https://www.government.se/government-of-sweden/ministry-of-health-and-social-affairs/
	https://platz.se/coronavirus/
Switzerland	www.bag.admin.ch
	https://www.bag.admin.ch/bag/de/home/krankheiten/ausbrueche-epidemien-pandemien/aktuelle-ausbrueche-epidemien/novel-cov/situation-schweiz-und-international.html
Taiwan	www.cdc.gov.tw
	https://www.cdc.gov.tw/Bulletin/List/MmgtpeidAR5Ooai4-fgHzQ?page=60
Tanzania	https://www.moh.go.tz/en/
	https://www.moh.go.tz/en/covid-19-info

Thailand	https://ddc.moph.go.th/viralpneumonia/eng/index.php
	https://ddc.moph.go.th/viralpneumonia/eng/news.php
	https://ddc.moph.go.th/viralpneumonia/eng/situation.php
Timor-Leste	www.noticias.sapo.tl
Togo	www.covid19.gouv.tg
Trinidad and Tobago	http://www.health.gov.tt/
	http://www.health.gov.tt/sitepages/default.aspx?id=293
Tunisia	http://www.santetunisie.rns.tn/fr/
	http://coronavirus.rns.tn/
Turkey	https://covid19.saglik.gov.tr/TR-68443/covid-19-durum-raporu.html
	https://covid19.saglik.gov.tr/TR-66935/genel-koronavirus-tablosu.html
Turks and Caicos Islands	www.gov.tc
	https://www.gov.tc/moh/coronavirus/
UAE	https://www.mohap.gov.ae/en/Pages/default.aspx
Ukraine	www.moz.gov.ua
	https://moz.gov.ua/novini?page=39
United Kingdom	https://coronavirus.data.gov.uk/
Uruguay	www.gub.uy
	https://www.gub.uy/sistema-nacional-emergencias/comunicacion/noticias/informacion-interes-actualizada-sobre-coronavirus-covid-19-uruguay
Uzbekistan	www.coronavirus.uz
Venezuela	www.minci.gob.ve
Vietnam	https://ncov.moh.gov.vn/
Yemen	https://reliefweb.int/report/yemen/ministry-health-aden-confirms-first-case-covid-19-yemen-enar
Zambia	www.mwebantu.com
	https://www.mwebantu.com/category/covid-19/
USA	_
	www.nj.gov
	https://coronavirus.illinois.gov/s/
	www.chicago.gov
	www.mass.gov
	www.health.pa.gov
	https://floridahealthcovid19.gov/
	https://coronavirus.maryland.gov/
	www.dph.georgia.gov
	www.ldh.la.gov
	www.in.gov
	https://covid19.colorado.gov/
	www.doh.wa.gov
	https://cv.nmhealth.org/
	www.nh.gov
	www.health.hawaii.gov
	www.covid19india.org
	https://health.ny.gov/

	https://www.dhhs.vic.gov.au/
	www.health.nsw.gov.au
	www.covidlive.com.au
International Organizations and others	https://www.who.int/
	https://www.who.int/emergencies/diseases/novel-coronavirus-2019/situation-reports
	https://www.who.int/news-room/releases/10
	https://covid19.who.int/
	https://www.who.int/csr/don/archive/disease/novel_coronavirus/en/
	https://www.paho.org/en/barbados-and-eastern-caribbean-countries
	https://www.paho.org/en/covid-19-situation-reports?topic=All&d%5Bmin%5D=&d%5Bmax%5D=&page=1
	https://www.paho.org/en/ecc-covid-19-situation-updates?topic=All&d%5Bmin%5D=&d%5Bmax%5D=&page=4
	https://www.cdc.gov/
	https://www.cdc.gov/coronavirus/2019-ncov/whats-new-all.html
	https://www.nih.gov/
	https://www.nih.gov/coronavirus-covid-19-news-releases?page=6
	https://www.paho.org/en
	https://www.who.int/emergencies/diseases/novel-coronavirus-2019/interactive-timeline
	http://www.emro.who.int/index.html
	http://www.emro.who.int/health-topics/corona-virus/situation-reports.html
	https://www.who.int/southeastasia
	who.int/emergencies/diseases/novel-coronavirus-2019/media-resources/news
	https://www.ecdc.europa.eu/en
	https://www.ecdc.europa.eu/en/covid-19/situation-updates
